# Popular Fallacies

**Published:** 1880-03

**Authors:** 


### POPULAR FALLACIES.

It is not true that sugar and candies are of themselves injurious to the teeth or the health of those who use them; so far from it, they are less injurious than any of the ordinary forms of food when employed in moderation.

Any scientific dentist will tell you, that the parts of teeth most liable to decay, are those which afford lodgment to particles of food; such particles being decomposed by moisture and heat, give out an acid, which will corrode steel as well as teeth; but pure sugar, and pure candies are wholly dissolved, there is no remnant to be decomposed to yield this destructive acid; we remember now no item of food which is so perfectly dissolved in the mouth as sugar and candy. When visiting the sugar plantations of Cuba, the attention was constantly arrested by the apparently white and solid teeth of the negroes who superintended the process of cane grinding; they drank the cane-juice like water, there was no restraint as to its use, and the little urchins playing about, would chew the sugar-yielding cane by the hour. It is much the same in Louisiana, where the shining faces and broad grins of the blacks are equally indicative of exuberant health and "splendid teeth."

How does it happen then that there should be "the prevalent belief" that sugar and sugar-candy destroy the teeth and undermine the health? Perhaps the most correct reply is *Tradition*, the father of a progeny of errors in theory and practice; of errors in doctrine and example, "too tedious to mention."

One of the common faults of the times is an indisposition to investigate on the part of the masses. We take too much for granted. A very common answer to a demand for a reason for a time honored custom, is "*Why, I have heard it all my life. Don't everybody say so?*"

It would be a strange contradiction in the nature of things, if sugar and candy in moderation, should be hurtful to the human body in any way, for sugar is a constituent of every article of food we can name; there is not a vegetable out of which it cannot be made, not a ripe fruit in our orchards which does not yield it in large proportions, and it is the main constituent of that "*milk*" which is provided for the young of animals and men all over the world. Perhaps the child has never lived which did not love sweet things beyond all others; it is an instinct, a passion, not less universal, than the love of water. A very little child can be hired to do for a bit of sugar what nothing else would. The reason of this is, that without sugar, no child could possibly live, it would freeze to death; it is the sugar in its food which keeps it warm, and warmth is the first necessity for a child.

But to use this information intelligently and profitably, it must be remembered that sugar is an artificial product, is a concentration, and that, if used in much larger proportions than would be found in our ordinary food, as provided by the beneficent FATHER of us all, we will suffer injury. We should never forget, that the immoderate use of any thing is destructive to human health and life, if persevered in. The best general rules to be observed are two:

First, Use concentrated sweets at meal times only.

Second, Use them occasionally, and in moderation.

